# Optic Disc Pit Maculopathy: One-Year Outcomes of Pars Plana Vitrectomy with Foveal Sparing Inverted Internal Limiting Membrane Flap

**DOI:** 10.7759/cureus.14057

**Published:** 2021-03-23

**Authors:** Palmeera D'souza, Shishir Verghese, Ratnesh Ranjan, Karan Kumarswamy, Veerappan R Saravanan, George J Manayath, Venkatapathy Narendran

**Affiliations:** 1 Department of Retina and Vitreous, Aravind Eye Hospital and Postgraduate Institute of Ophthalmology, Coimbatore, IND

**Keywords:** optic disc pit, optic disc pit maculopathy, inverted internal limiting membrane flap, ilm flap, foveal sparing internal limiting membrane peeling, foveal sparing ilm peeling

## Abstract

Purpose

To evaluate the anatomical and visual outcomes in optic disc pit maculopathy following pars plana vitrectomy (PPV) with inverted internal limiting membrane (ILM) flap

Methods

Retrospective interventional case series of 10 patients diagnosed with serous macular detachment secondary to optic disc pit who underwent PPV with inverted ILM flap and were followed up for a year.

Results

A p-value of less than 0.05 was defined as statistically significant. The mean age of patients was 27.2 ± 10.6 years, preoperatively the mean best-corrected visual acuity of the logarithm of the minimum angle of resolution was 0.91 ± 0.42 (approximate Snellen equivalent 20/162), which improved to the logarithm of the minimum angle of resolution of 0.58 ± 0.29 (approximate Snellen equivalent 20/76) at end of one year, (p=0.008). The mean central macular thickness was 804.9 ± 294.1 m which improved to 273.4 ± 102.54 m, (p=0.002). After surgery, at end of one year, 60% of patients (6/10) had 15- or more-than-15-letter improvement of vision on Early Treatment Diabetic Retinopathy Study (ETDRS) visual acuity testing, 20% (2/10) gained a 10-letter improvement and 20% (2/10) retained the same vision.

Conclusion

PPV with inverted ILM flap can be considered as a good approach for the management of serous macular detachment secondary to optic disc pit and produce good anatomical and visual results at one year with stabilization of the disease.

## Introduction

Optic disc pit (ODP) is a rare congenital anomaly of the optic nerve head (ONH) appearing as a small hypo-pigmented grey-white depression usually located in the temporal part of the optic disc [1]. ODPs are thought to result from the failed closure of fetal fissure during embryogenesis. The incidence of ODP has been reported to be 1 in 11,000 cases [2] without gender predilection with only 15% of cases being bilateral [3, 4]. ODPs are usually asymptomatic, however, 25-75% of cases present with defective vision if associated with ODP-related maculopathy (ODPM) [2, 4]. ODPM is characterized by the presence of retinal schisis and serous macular detachment (SMD) [5-7]. Other retinal findings in long-standing ODPM include cystoid macular edema (CME), lamellar macular holes (LMH), full-thickness macular hole (FTMH), and retinal pigment epithelium (RPE) atrophy, which is usually associated with a poor visual acuity of 20/200 or worse [8-10]. The exact pathogenic mechanism of ODPM remains unclear, however, it has been proposed that vitreous or cerebrospinal fluid (CSF) may be the originof the fluid [11-13].

A myriad of treatment options is available for ODPM including conservative management, laser photocoagulation [14,15], intravitreal gas tamponade [16,17], macular buckling surgery [18], and pars plana vitrectomy (PPV) with or without plugging of ODP [19-23]. Various substances used for ODP plugging include internal limiting membrane (ILM) flap [22, 23], scleral autograft [24], and autologous fibrin and fibrin sealants [25, 26]. Studies have shown variable outcomes following different treatment modalities. PPV has been shown to cause good anatomical and functional outcomes in more than 50% of cases [27]. PPV with OPD plugging using ILM flap is another described technique to have favorable outcomes, but there is a risk of FTMH formation, especially where the retina is extremely thinned out [28]. The use of fovea sparing inverted ILM flap for ODP plugging has shown promising results in anecdotal reports [10, 22, 23, 28]. In literature, there is a lack of large case series of ODPM undergoing PPV with fovea sparing inverted ILM flap and its long-term outcomes.

The aim of this study is to describe the long-term outcomes of ODPM management with the modified surgical technique of vitrectomy with ODP plugging using fovea sparing inverted ILM flap.

## Materials and methods

This retrospective study included all consecutive patients of ODPM who underwent PPV with fovea sparing inverted ILM flap and had a minimum follow-up of one year between January 2016 to December 2019 in a tertiary eye care hospital in south India. The study was conducted according to the tenets of the Declaration of Helsinki. An ethics committee approval was obtained for performing the study (IHEC Number: 21/022).

All patients were diagnosed with ODPM based on clinical examination with slit-lamp bio-microscopy, fundus examination, and spectral-domain optical coherence tomography (SD-OCT) (Spectralis OCT, Heidelberg Engineering, Heidelberg, Germany) imaging. Pre-operative Snellen’s best-corrected visual acuity (BCVA) and intraocular pressure (IOP) were noted. Informed written consent was taken for surgical intervention.

All surgeries were performed by either of two surgeons (V.R.S, P.D.) in the affected eyes of patients with ODPM following a standard surgical technique. Twenty-five gauge 3-port PPV was done followed by induction of posterior vitreous detachment (PVD). Brilliant blue dye (Ocublue plus 0.05% w/v, Aurolab, Madurai, India) was used to stain the ILM. A 25G pick was used to raise the ILM flap in an area superonasal to fovea followed by a fovea sparing ILM peeling up to the temporal edge of the ONH using ILM forceps. A small drop of 1.4% viscoelastic sodium hyaluronate was injected slowly over the disc pit using a straight cannula. The ILM flap was inverted and stuffed over the ODP. A fluid air exchange (FAE) was done using a silicone-tipped flute needle from the nasal side of ONH. A non-expansive concentration of 20% sulphur hexafluoride (SF6) gas or 14% perfluoro-propane (C3F8) was injected. Postoperatively, the prone position was advised for two weeks.

The post-operative examination was done on day one, at two weeks, and monthly thereafter for six months, followed by three monthly follow up. Postoperative work-up during every follow-up included BCVA and IOP measurement, clinical examination of anterior segment and fundus. Postoperative OCT imaging was started only from the one-month follow-up once media became clear enough after partial absorption of intraocular gas.

Pre- and postoperative OCT images were analyzed to assess the various features of ODPM. The pre-operative assessments included central macular thickness (CMT), the type of macular elevation (retinal schisis and SMD), the existence of LMH or FTMH, the location of the ODP on the ONH, the communication between the ODP and retinal or subretinal fluid, and the presence of any vitreoretinal interface abnormality such as vitreomacular traction (VMT) or vitreous strands on the ODP. The status of the ellipsoid zone (inner segment/outer segment junction) of the photoreceptors layer was studied in the pre- and postoperative OCT images. The ellipsoid zone without any discontinuity was described as “intact”, with a discontinuity of <200 microns as “disrupted”, and with a gap ≥200 microns as “absent”. The postoperative macular thickness for patients who developed full-thickness macular hole was calculated from the base of the hole to the edge of the wall of the hole using the measurement software in the OCT.

All data was entered on an Excel spreadsheet (Microsoft Corporation, Redmond, WA, USA). Statistical analysis was performed using SSPS 20.0 statistical software (IBM Corp, Armonk, NY, USA). For statistical analysis, Snellen’s BCVA was converted to logarithm of the minimum angle of resolution (logMAR). 

Significant visual improvement was defined as equal to or more than 15-letter improvement on the ETDRS visual acuity testing. For the comparative and correlational analysis, various factors measured preoperatively and postoperatively at six months and at one year were included and were analyzed using the Wilcoxon matched-pairs test. A p-value of less than 0.05 was defined as statistically significant.

## Results

This retrospective study included 10 eyes of 10 patients (five males and five females). The mean age of patients enrolled was 27.20 ± 10.06 years, range (11-43 years). The mean BCVA was logMAR 0.91 ± 0.42 (range 0.60-2.0) [approximate Snellen equivalent 20/163 (range 20/80 - 20/2000)]. The preoperative mean CMT was 804.9 ± 294.1 mm (range 422-1303 mm). In 90% of patients (9/10), macular elevation was attributed to SMD and/or retinal schisis. Multilevel fluid, including inner and outer retinal schisis, as well as SMD, was present in 70% (7/10) of cases. In all eyes, ODP was located at the temporal edge of ONH. An obvious communication between the ODP and macular elevation was observed only in two cases (20%). Baseline vitreomacular traction (VMT) was only seen in one case (10%), while the presence of vitreous strands emerging from the ODP was seen in two cases (20%). Six patients (60%) had outer LMH, while FTMH was not present in any patient. The ellipsoid zone was intact in three patients (30%), absent in six patients (60%), and disrupted in one patient (10%).

Surgical intervention resulted in a significant reduction of retinal elevation with a mean CMT of 341.5 ± 116.38 mm (p=0.002) at six months follow up. At one-year follow-up, mean CMT decreased further to 273.4 ± 102.54 (p=0.008) with complete resolution of maculopathy in seven patients (70%) and shallow residual SMD in three patients (30%).

A statistically significant improvement in mean BCVA was noted at six months logMAR 0.63 ± 0.26 (approximate Snellen equivalent 20/85); p=0.008, and at one-year logMAR 0.58 ± 0.29 (approximate Snellen equivalent 20/76); p=0.008. Descriptive analysis showed significant visual improvement of 15 or >15-letter ETDRS in six patients, a 10-letter improvement in two patients and no change in BCVA in two patients.

At one-year follow up, ellipsoid zone was found intact in six patients (60%), disrupted in two patients (20%), and absent in two patients (20%). A weak correlation existed between the grades of ellipsoid zone integrity and final BCVA at one year (Spearman’s r=0.592, p=0.0711). No other complication like cataract and IOP elevation was observed in the operated eyes at one year follow up. 

**Figure 1:**
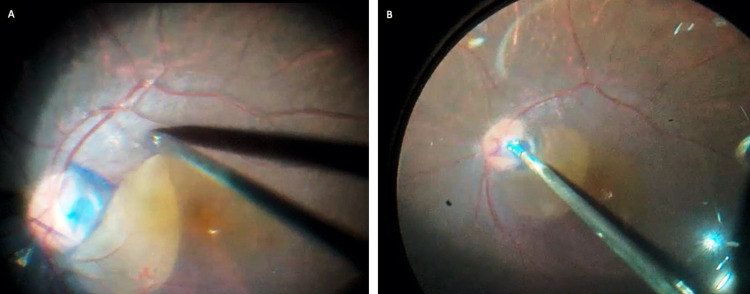
Patient 1 (RE). Intraoperative picture (surgeon’s view) demonstrating a fovea sparing ILM peel in the right eye with optic disc pit maculopathy. (A) Figure showing grasping and peeling of the ILM (sparing fovea) with a 25G Max-grip forceps (Grieshaber, Alcon, Fort Worth, TX, USA); (B) Plugging of inverted ILM flap over the optic disc pit during a fluid air exchange RE – right eye, ILM – internal limiting membrane

**Figure 2:**
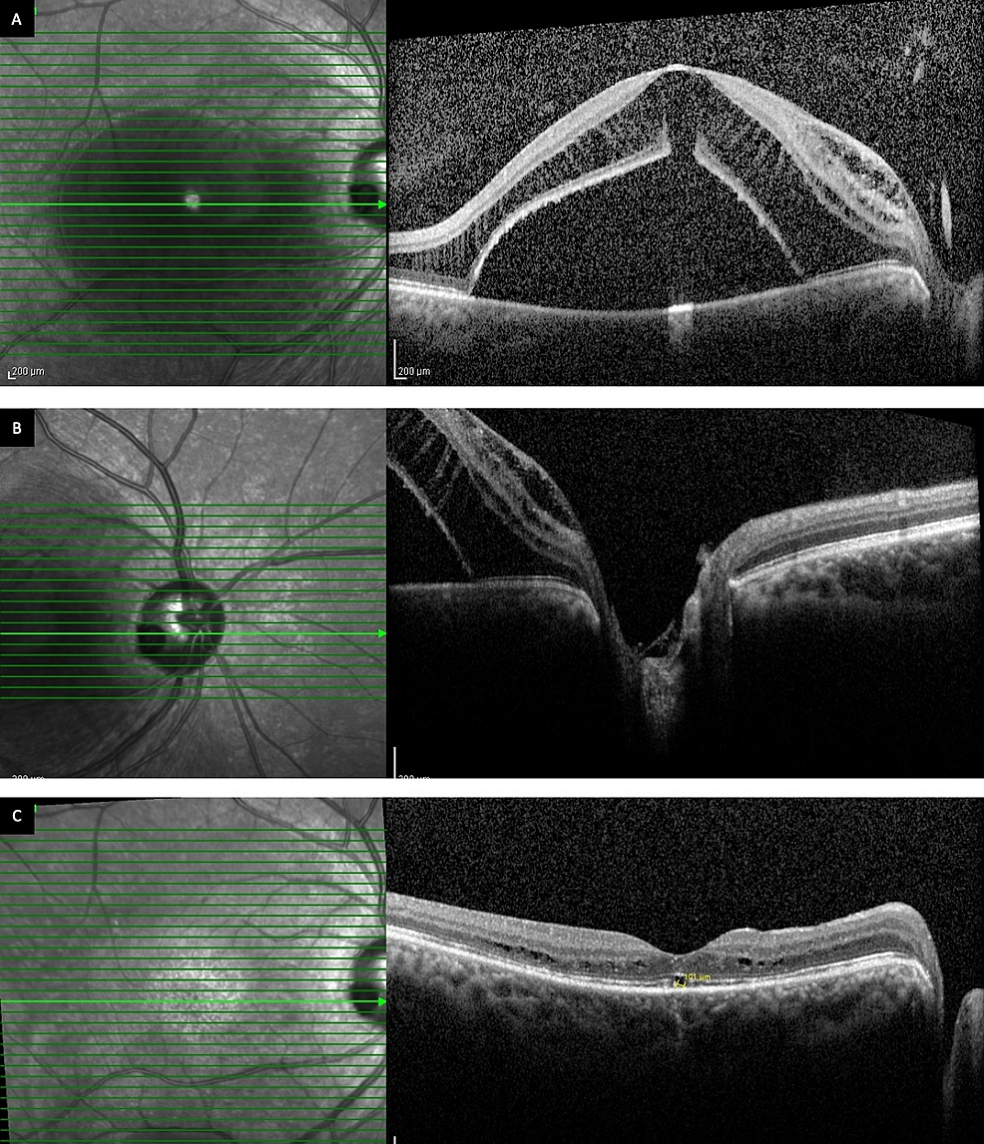
Patient 1 (RE): (A) Preoperative OCT through the fovea showing maculopathy with dome-shaped SRF with inner and outer retinal layer schisis, and outer lamellar hole; (B) Preoperative OCT of the optic disc showing optic disc pit; [C] Post-operative OCT at one year showing complete SRF resolution with minimal intra retinal cystic spaces. OCT – ocular coherence tomography, RE – right eye, SRF – subretinal fluid

**Figure 3:**
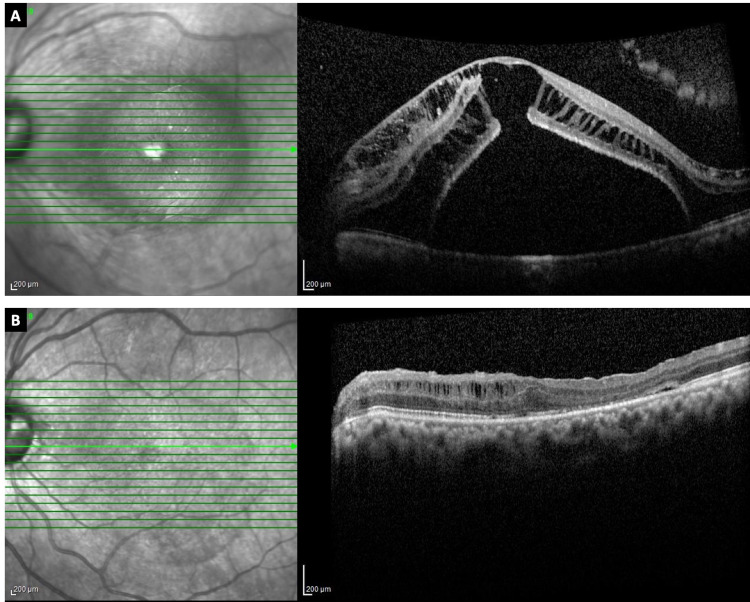
Patient 7 (LE). (A) Preoperative OCT through the fovea showing schisis of inner and outer retinal layers; (B) Postoperative OCT at one year showing resolution of subretinal fluid and minimal inner retinal schisis nasally. OCT – ocular coherence tomography; LE – left eye

**Figure 4:**
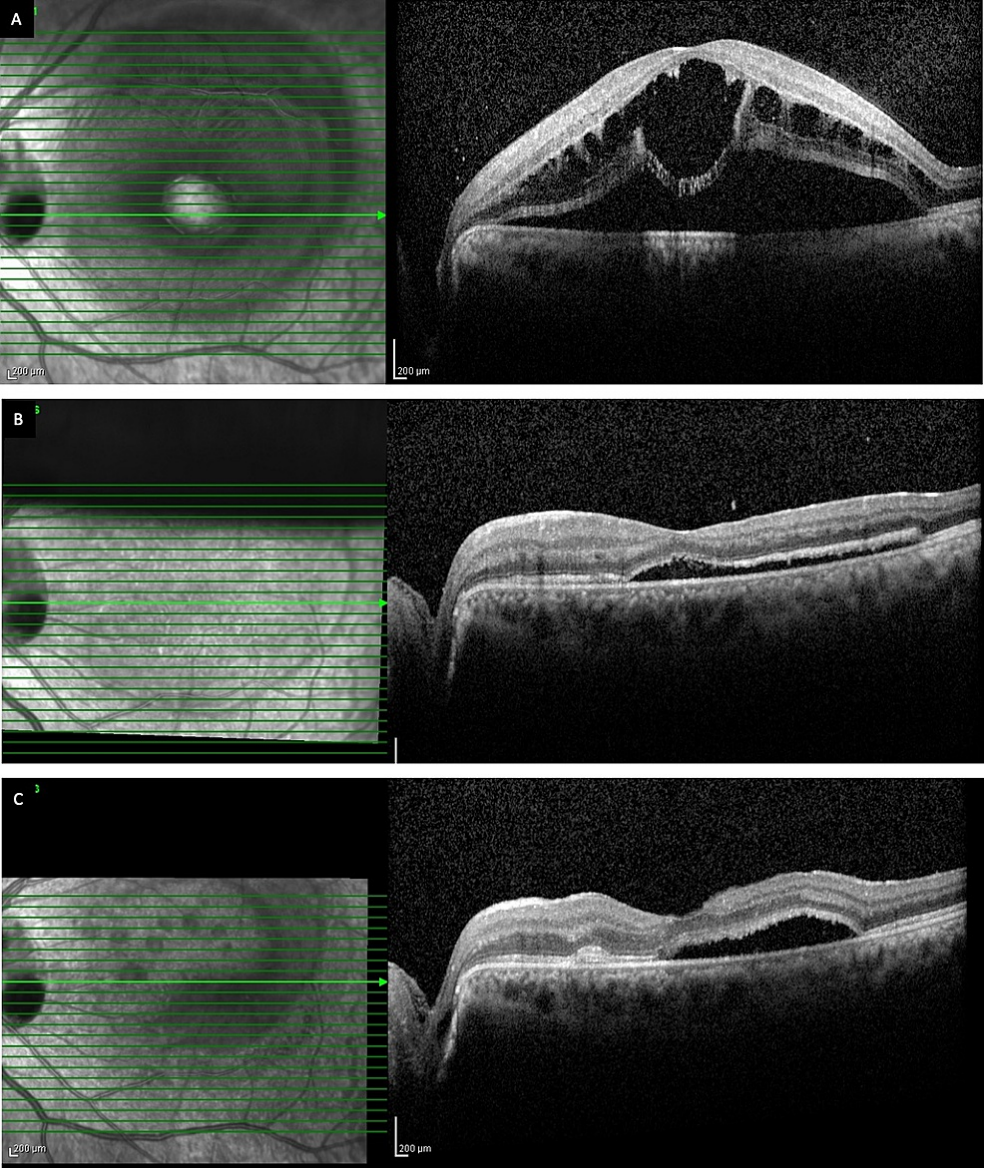
Patient 6 (LE). Preoperative OCT through the fovea of patient 6 who presented with a visual acuity of counting fingers 2 meters (Snellen equivalent 20/2000). (A) macula showing dome shaped SRF with outer retinal layer schisis and outer retinal cyst; (B) Postoperative OCT at one month showing significant SRF reduction; (C) Postoperative OCT at 1 year showing persistent SRF (partial resolution) with mild photoreceptor elongation (brush border pattern), visual acuity improved to 20/125. OCT – ocular coherence tomography; SRF – subretinal fluid

The demographic pre- and postoperative data of all patients are shown in Table 1. Intraoperative photograph and OCT images of selected cases are shown in Figures 1-4.

**Table 1:** Pre and post-operative patient characteristics VA – Visual acuity, CMT – Central Macular Thickness, SMD – Serous macular detachment, CMT – Central macular thickness, FTMH – Full-thickness macular hole, PR layer – Photoreceptor layer, A – Absent, P – Present, D – Disruption, OLH – Outer lamellar hole, ETDRS – Early Treatment Diabetic Retinopathy Study

Patient No	Age at presentation	Sex	Baseline				One year				Visual improvement/worsening (ETDRS letters)
			VA (logMAR) (Snellen Equivalent)	OCT			VA (logMAR) (Snellen Equivalent)	OCT			
				CMT (m)	Maculopathy features	PR layer		CMT (m)	Maculopathy features	PR layer	
1	23	F	0.6 (20/80)	660	SMD + Schisis	D	0.3 (20/40)	180	Complete Resolution	D	15 letter improvement
2	16	M	0.8 (20/125)	747	SMD + Schisis	A	0.5 (20/63)	401	Partial resolution	P	15 letter improvement
3	11	F	0.8 (20/125)	422	SMD	P	0.3 (20/40)	244	Complete resolution	P	25 letter improvement
4	24	M	0.6 (20/80)	741	SMD + Schisis	A	0.3 (20/40)	134	Complete resolution	P	15 letter improvement
5	24	M	1 (20/200)	497	SMD + Schisis	A	1 (20/200)	181	Complete resolution	A	Status quo
6	31	F	2 (20/2000)	975	Schisis	P	0.8 (20/125)	325	Partial resolution	P	45 letter improvement
7	36	F	0.5 (20/63)	850	SMD + Schisis	A	0.3 (20/40)	251	Complete resolution	D	10 letter improvement
8	39	M	1 (20/200)	611	SMD + Schisis	A	1 (20/200)	461	Partial resolution	A	Status quo
9	25	M	0.8 (20/125)	1243	SMD + Schisis	P	0.5 (20/63)	313	Complete resolution	P	15 letter improvement
10	43	F	1 (20/200)	1303	SMD + Schisis	A	0.8 (20/125)	244	Complete resolution	P	10 letter improvement

## Discussion

This study describes a larger series of ODPM cases managed with the modified surgical technique of PPV and plugging of ODP with fovea sparing inverted ILM flap. The long-term outcomes suggest that surgical intervention results in anatomic and functional improvement. 

Similar to previous studies, ODP was unilateral and located in the temporal half of ONH in all patients. ODP was seen equally among male and female as reported previously. All patients with ODPM in this study presented with defective vision. We analyzed the pre- and postoperative integrity of the photoreceptor ellipsoid zone to correlate it with the final visual outcome. There was only a weak correlation noted in our study. No previous ODPM study has analyzed this correlation except that conducted by Theodossiadis et al. where they managed ODPM surgically with macular buckle and found a significant correlation between the photoreceptor layer integrity and postoperative BCVA at all follow-ups. [18].

Several theories concerning the pathogenesis of ODP maculopathy have been proposed over the years with the exact mechanism not being clarified. Among the most prevailing theories are those that support the origin of fluid from the vitreous cavity or the subarachnoid space. [11-13] In the former, it has been proposed that vitreous exerts traction on the optic disc and macula which causes a negative pressure gradient leading to fluid entry to the sub-macular space through the ODP [11]. Only one patient in our study had VMT seen on OCT similar to the case series published by Rizzo et al. [21]. In the latter, it has been postulated that the CSF flows directly into the sub-macular space or into the retina through a direct communicating tract from subarachnoid space via the ODP defect. In our study, no obvious communicating channel was seen. In most cases, the fluid follows the pattern as described by Lincoff et al., where fluid from the ODP first creates a schisis-like separation of the inner retina and then reaches the sub-retinal space, creating a macular neuro-epithelial detachment [7]. Spontaneous resorption of fluid is possible in 25% of patients according to Gass [1]. In the remaining patients, the chronic nature of maculopathy causes progressive deterioration of macular structures leading to a reduction in vision and thereby warranting treatment for this condition.

Many surgical techniques and their combinations have been described by investigators with variable outcomes. Studies have shown promising results after PPV for the treatment of ODPM resulting in both retinal re-attachment and visual improvement. A higher anatomical success rate (50-95%) and functional visual acuity improvement (> 50%) have been reported when PPV is used in combination with additional procedures such as ILM peeling, peripapillary laser photocoagulation, and gas tamponade [19-23]. One of the commonly performed additional surgical step described in the literature is creating a flap by ILM peeling used to plug the ODP. In the majority of studies describing this technique, the ILM peeling was performed across the fovea which increases the risk of foveal de-roofing and FTMH formation. 

Recently, a modified technique of fovea sparing ILM peeling has been described in a few case reports [22, 23, 28, 29]. In this study, we have evaluated this modified technique of PPV and ODP plugging with fovea sparing ILM flap in a larger series of 10 patients. The rationale of using this technique include PPV negating the vitreous traction at the optic disc, fovea sparing ILM peeling reducing the risk of foveal de-roofing and plugging of ODP with inverted ILM flap, blocking the communication between the pit and sub- or intra-retinal space. Additionally, PPV with ILM peeling helps to remove anteroposterior and tangential vector forces helping in the closure of the communicating channel.

This modified surgical technique resulted in significant improvement in anatomical and visual outcomes in terms of fluid resolution and reduction in the central macular thickness at six months, which was maintained till the one-year follow up in this study. Similar successful anatomical outcomes have been reported following this technique for ODPM in previous reports [22, 28, 29]. Dhiman et al. [28] and Roy et al. [29] used this technique as the primary procedure in one patient each, while Hara et al. [22] performed this technique as a secondary procedure after a failed primary surgery. All cases were reported to have successful anatomical outcome with a suboptimal visual outcome [22, 28]. In our study, 80% of patients showed a 10 and >10 ETDRS letter improvement (Table 1) in Snellen’s BCVA at one year, with overall statistically significant visual improvement, p=0.008. The chronicity of sub-retinal and intra-retinal fluid might be accounted for the suboptimal functional outcome in two of our patients of ODPM. The presence of multilayer fluid including inner and outer retinal schisis and SMD are suggestive of the chronicity of ODPM and considered as a poor prognostic factor for the postoperative outcomes [30]. Steel et al. found that ODPM cases with multilayer intra-retinal and sub-retinal fluid were less likely to have visual success following surgical intervention [30]. We also observed only a weak correlation between the final BCVA and the integrity of the ellipsoid zone.

Fovea involving ILM peeling carries a higher risk of FTMH formation especially in cases of ODPM with extremely thinned out retina. In a study by Shukla et al., though good surgical outcomes were recorded in terms of restoration of macular anatomy following vitrectomy with ILM peeling and gas tamponade, more than half of the patients (4/7) developed FTMH postoperatively. The authors attributed the high incidence of FTMH to the peeling of ILM over thinned out retina [9]. The probable mechanism of FTMH formation in these cases could be a subclinical de-roofing of fovea during PVD induction due to strong vitreoretinal attachment at the thinned-out fovea, which expands later to form FTMH. Another possible mechanism of macular hole formation is progressive schisis as seen during the natural course of advanced ODPM. Theoretically, the modified surgical technique used in this study provides the advantage of avoiding foveal de-roofing during ILM peeling and consequently reducing the risk of FTMH formation. In our series, FTMH was not noted preoperatively, intraoperatively or in the immediate postoperative period. Therefore, this modified technique eliminates the risk of FTMH formation including the risk of iatrogenic hole formation.

Despite being the largest series of this modified surgical technique for ODPM, the small sample size and non-comparative nature of the study are the main limitations of this study. 

## Conclusions

The modified technique of PPV and plugging of ODP using fovea sparing inverted ILM flap for the treatment of ODPM results in good anatomical and functional outcomes. Surgical intervention may also help in the long-term stabilization of the disease. Larger randomized comparative studies are required to understand the pathogenesis and the best surgical approach in eyes with ODPM.
